# Case Report: Overlap syndrome of anti–NMDA receptor encephalitis and MOG-associated disease in a pediatric patient—literature insights

**DOI:** 10.3389/fimmu.2026.1694771

**Published:** 2026-03-10

**Authors:** Dhouha Krir, Maha Jamoussi, Ahlem Ben Hmid, Hanene Ben Rhouma, Sonia Nagi, Yousr Galai, Samar Samoud, Hédia Klaa, Ichraf Kraoua, Mélika Ben Ahmed, Imen Zamali

**Affiliations:** 1 Faculty of Medicine of Tunis, University of Tunis El Manar, Tunis, Tunisia; 2 Department of Child and Adolescent Neurology, National Institute Mongi Ben Hmida of Neurology, Tunis, Tunisia; 3 Department of Clinical Immunology, Pasteur Institute of Tunis, Tunis, Tunisia; 4 Laboratory of Transmission, Control and Immunobiology of Infection, Pasteur Institute of Tunis, Tunis, Tunisia; 5 Research Laboratory and Neuroradiology Department, National Institute Mongi Ben Hmida of Neurology, Tunis, Tunisia

**Keywords:** Anti–NMDA receptor encephalitis, myelin oligodendrocyte glycoprotein–associated disease, overlap syndrome, autoimmune neurological disorders, pediatric neurology

## Abstract

Myelin oligodendrocyte glycoprotein antibody disease (MOGAD) and anti-N-methyl-D- aspartate receptor (NMDAR) encephalitis pediatric cases are especially challenging due to phenotypic variability, limited literature, and the absence of standardized treatment protocols. We present the first documented African case of pediatric MOG and NMDAR overlapping syndrome (MNOS), with a review of all pediatric MNOS cases reported thus far in the literature. A previously healthy seven-year-old boy developed rapid-onset sleep disturbances, neuropsychiatric symptoms, multiple cranial nerve palsies, and hyperkinetic movements. Serological and cerebrospinal fluid (CSF) analyses confirmed dual positivity for anti- MOG and anti- NMDAR antibodies. The patient responded favorably to first line immunotherapy with intravenous immunoglobulin and corticosteroids, showing marked clinical improvement by the six-month follow-up. A corticosteroid taper was initiated thereafter. At fourteen-month follow-up, he had a second episode with a MOGAD- associated cerebral cortical encephalitis phenotype. Antibody screening confirmed persistent dual positivity for both anti-NMDAR and anti-MOG antibodies. The clinical outcome was favorable following first-line immunotherapy combined with oral immunosuppressants. This case highlights the uniqueness of this entity, where antibody dynamics are closely tied to the clinical course.

## 1 Introduction

Autoimmune encephalitis is a relatively frequent condition with an incidence estimated at 5 to 8 cases per 100–000 individuals (1). Significant advances in immunology, particularly the identification of neuronal auto-antibodies (abs), have profoundly impacted the clinical management of various neurological syndromes.

Anti-N-methyl-D-aspartate receptor (NMDAR) encephalitis is one of the first described autoimmune neurological disorders. Approximately 6% of patients with anti-NMDAR encephalitis may present either concurrently or consecutively with a demyelinating syndrome (2, 3), especially in those targeting the water channel aquaporin-4 immunoglobulin G (AQP4- IgG) antibodies (3, 4). The concurrent presence of myelin oligodendrocyte glycoprotein (MOG) and NMDAR abs is a rare clinical phenomenon, with an estimated incidence of less than 10% at the onset of the disease (5).

With the increasing number of similar studies published, it has become evident that the prevalence of these coexisting anti-NMDAR-IgG and MOG-IgG may be underestimated. Their clinical features and therapeutic challenges remain inadequately characterized. To our knowledge, only sixty-three cases described thus far in children, though the exact number remains uncertain (3, 6–17). Herein we report the first African pediatric case of a child with a demyelinating syndrome where both anti-NMDAR and anti-MOG antibodies have been detected and comprehensively describing the MOG and NMDAR overlapping syndrome (MNOS), highlighting its clinical, radiological, and immunological features.

## 2 Case description

### 2.1 Initial presentation and acute phase

A seven-year-old boy, originating from Tunisia, with no familial or personal medical history and no previous or current medication, presented with a two-month history of reduced visual acuity, right-sided ocular misalignment, headaches, and drowsiness. The patient’s condition progressively worsened, with the development of marked sleep disturbances, disinhibited behavior, psychomotor regression and acute abnormal movements, requiring referral to the Child and Adolescent Neurology Department of our specialized center in January 2024.

Neurological examination revealed right hemiparesis with bilateral pyramidal tract signs, static cerebella ataxia, multiple cranial nerve involvement including visual impairment, a right-sided divergent strabismus with bilateral Ophthalmoplegia (bilateral limitation of verticality and adduction). The Clinical Assessment Scale in Autoimmune Encephalitis (CASE) score at admission was 9/27. Ophthalmologic examination revealed a visual acuity limited to light perception on the right eye with a visual acuity of 5/10 on the left eye. Both eyes demonstrated a sluggish pupillary light reflex and pale optic discs. Macular Optical Coherence Tomography (OCT) demonstrated significant thinning of the macular ganglion cell layer (GCL); papillary OCT revealed marked thinning of the temporal retinal nerve fiber layer (RNFL), indicative of bi-temporal optic nerve atrophy. We also noted cataplexy and generalized chorea.

### 2.2 Diagnostic workup and findings

**Figure 1:**
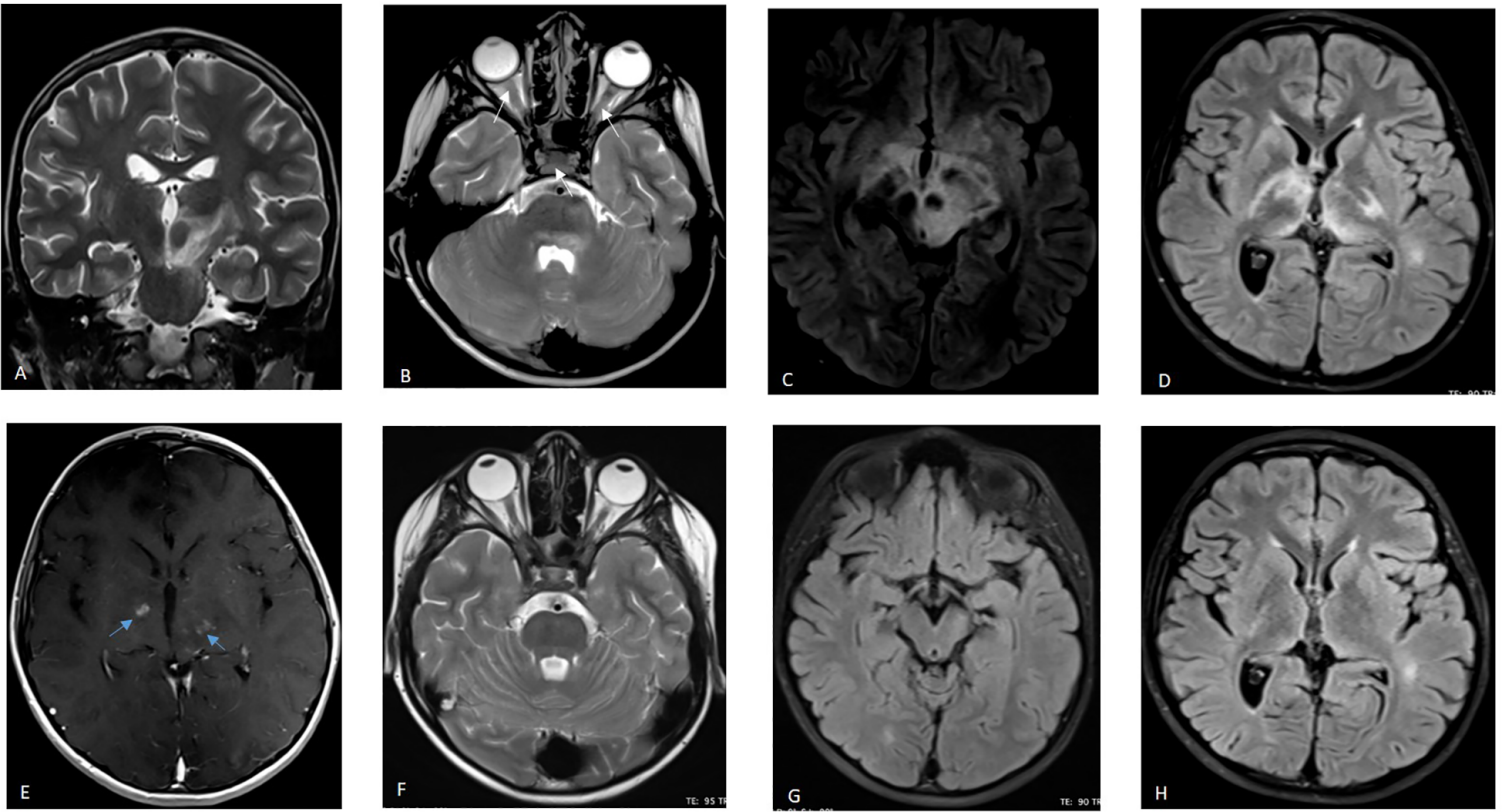
Brain MRI: coronal T2 weighted image **(A)**, axial T2 weighted image **(B)** and axial FLAIR weighted images **(C, D)**, show a poorly limited diencephalic infiltrative lesion extending into the brainstem, along with bilateral involvement of optic nerves and chiasm (white arrow). Axial T1-weighted image after gadolinium injection shows patchy bilateral contrast enhancement (blue arrows) in the basal ganglia **(E)**. Follow-up brain MRI at eight months: axial T2 weighted **(F)** and FLAIR-weighted images **(G, H)** show marked reduction of the brainstem and diencephalic hypersignal.

**Figure 2:**
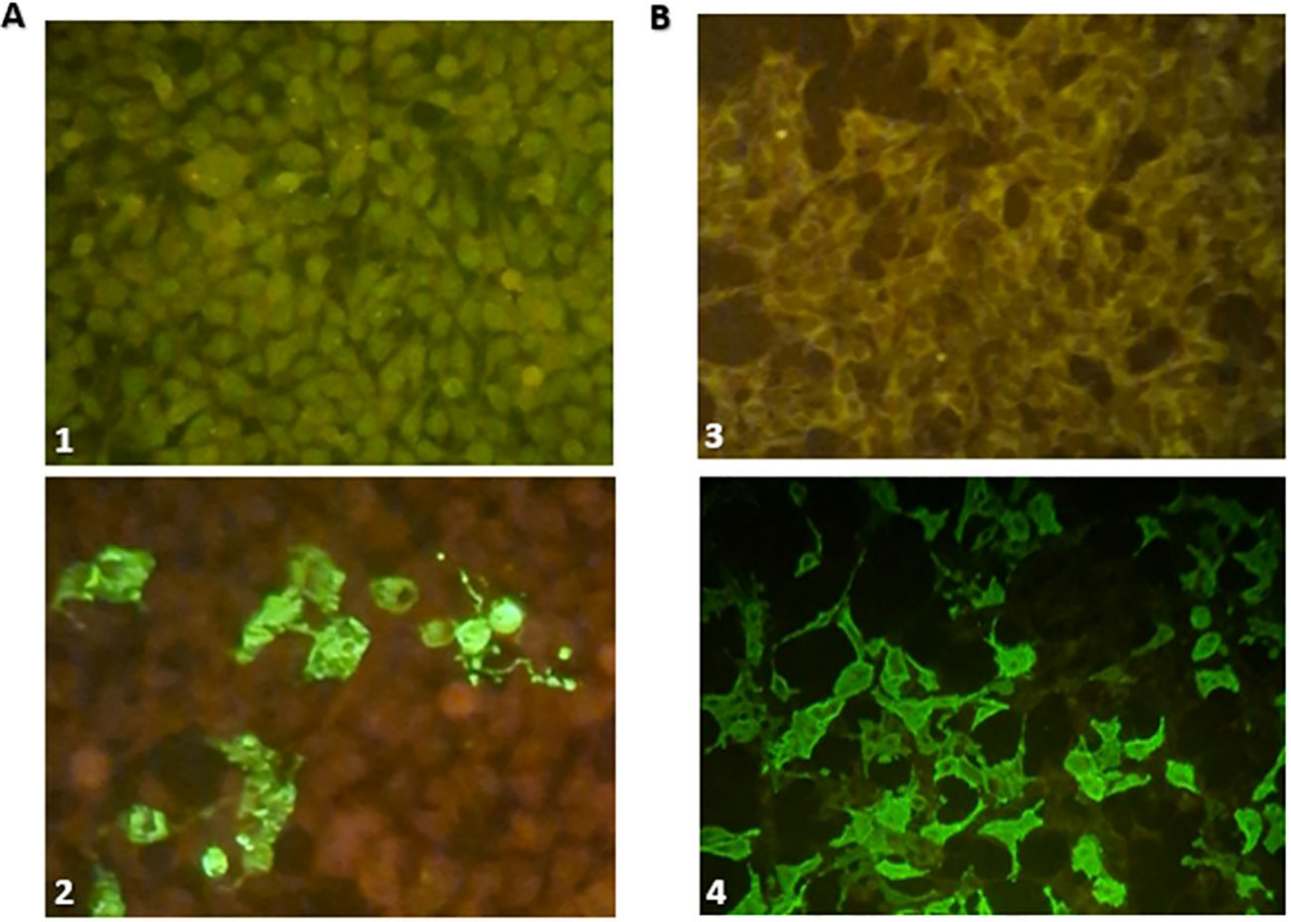
**(A)** Detection of NMDAR-IgG using indirect immunofluorescence by BIOCHIPs containing transfected cells (Euroimmun®) at 40× magnification (1) Negative control (2) Patient positive serum (++). **(B)** Detection of MOG-IgG using indirect immunofluorescence by FCBA (Euroimmun®) at 40× magnification (3) Negative control (4) Patient positive serum (1/100).

Brain Magnetic Resonance Imaging (MRI) demonstrated an extensive diencephalic infiltrative lesion extending into the brainstem, with bilateral anterior optic nerve and optic chiasm involvement, suggestive of MOGAD (**Figure 1**). Electroencephalography (EEG) revealed mild generalized slowing of background activity. Biochemical and microbiological analysis of cerebrospinal fluid (CSF) was unremarkable. Anti-MOG and anti- NMDAR antibodies were detected in both serum and CSF using a commercial fixed cell- based assay (CBA, Euroimmun^®^ AG, Lübeck, Germany). A threshold titer >1:10 was considered positive. The intensity of anti-NMDAR antibody positivity was graded semi- quantitatively on a scale of 1+ to 3+, with the number of crosses indicating the degree of reactivity. Anti-MOG antibodies were detected at titers of 1:100 in both serum and CSF, while anti-NMDAR antibodies showed a signal intensity of 2+ in both compartments (**Figure 2**). Repeat antibody testing confirmed the initial findings. Antibodies against Cv2, Ma2, Ri, Yo, Hu, recoverin, titin, SOX1, and amphiphysin were tested by the commercial immunoblot kit EUROLINE Paraneoplastic Neurological Syndromes 12 Ag (Euroimmun^®^) following the manufacturers’ instructions at serum dilution 1:100. Antibodies against GAD65 were detected using a commercialized enzyme-linked immunosorbent assay from Euroimmun^®^.

Isoelectric focusing (IEF) of CSF and serum proteins (Hydragel CSF Isofocusing kit, Sebia^®^, France) revealed mild intrathecal synthesis of oligoclonal immunoglobulins, characterized by CSF- restricted IgG oligoclonal bands (OCBs). Additionally, the IgG index was 0.86 without evidence of a significant blood-brain barrier disruption. No other abnormalities were detected on immunological testing. The paraneoplastic workup, including a chest, abdomen, and pelvis computed tomography scan, was negative except for a moderately elevated neuron-specific enolase (NSE) level of 31.35 ng/ml (reference range ≤ 17 ng/ml). Other tumor markers, such as carcinoembryonic antigen (CEA), CA 125, and CA 19- 9, were negative. MOGAD diagnostic criteria were met (18), including a core demyelinating event (Acute Disseminated Encephalomyelitis), supportive MRI features and the presence of IgG anti-MOG antibodies. NMDAR encephalitis was diagnosed based on the rapid onset of core clinical features (< 3 months) including abnormal behavior, speech dysfunction, movement disorders, associated with a slow EEG, OCBs on IEF of CSF and serum proteins, and the presence of anti-NMDAR-abs.

Additionally, alternative causes of encephalitis were systematically excluded. CSF analysis showed no evidence of bacterial infection, with negative Gram stain and culture; given this and the strong clinical and radiological suspicion for autoimmune encephalitis, viral polymerase chain reaction assays were not indicated. Serological testing excluded HIV as well as hepatitis B and C infections. AQP4-IgG testing was performed and was negative, helping to exclude NMOSD and supporting MOGAD as the demyelinating component of MNOS. A comprehensive autoimmune encephalitis antibody panel (including anti-LGI1, CASPR2, AMPAR1, AMPAR2, and GABABR) was also negative, reinforcing the specificity of the dual MOG- and NMDAR-antibody positivity in this case.

### 2.3 Treatment approach and initial response

Our therapeutic approach was aligned with the current recommendations of the European Pediatric MOG Consortium Consensus (19). Our first-line treatment with high-dose corticosteroids and intravenous immunoglobulin was consistent with European Pediatric MOG Consortium recommendations (19). Although the neurological presentation was severe and suggestive of an aggressive inflammatory encephalitis, escalation to stronger rescue immunosuppressive therapies such as rituximab was deliberately deferred, as the patient demonstrated a clear, early, and sustained clinical response to first-line therapy, with significant neurological improvement (CASE score reduced from 9/27 to 3/27 at six months) and radiological stabilization. Current literature supports reserving second-line immunotherapy for refractory disease, insufficient response to first-line treatment, or early relapse, rather than systematic upfront escalation, particularly in pediatric patients (41, 43).

Given the overlapping clinical features of MOGAD and NMDAR encephalitis, and the severity of neurological manifestations, an intensive first-line immunotherapeutic strategy was nevertheless adopted consisting of high- dose corticosteroids and intravenous immunoglobulin (IVIG) was initiated (0.4 g/kg/day) for 5 days, with a total of six courses within six months. Intravenous methylprednisolone was administered at a dose of 30 mg/kg/day for 5 days, followed by oral prednisone at a dose of 1.5 mg/kg/day. This approach was intended to achieve effective disease control while avoiding premature exposure to long-term or B-cell–depleting immunosuppression in a pediatric patient who was responding favorably to first-line therapy. The patient was discharged with a significant improvement in his visual acuity, ocular motility and sleep disturbances. At six-month follow-up, significant neurological improvement was noted, including full alertness, improved visual acuity and ocular motility, normal gait, and no sleep disturbances, with a CASE score of 3/27. He was left with persistent right third nerve palsy, manifesting as intermittent diplopia and divergent strabismus on the right side. Follow-up imaging conducted at eight months showed attenuation of initial hypersignal in the diencephalons and brainstem (**Figure 1**). Anti-MOG antibodies remained positive in the serum (1:100). Steroid tapering was therefore guided by a multifactorial and individualized assessment, guided by clinical improvement (resolution of sleep disturbances, behavioral changes, ocular motility deficit and motor signs, with stabilization of the CASE score), radiological regression of diencephalic and brainstem lesions on the 8-month follow-up MRI, and immunological monitoring showing persistent MOG-IgG positivity, which supported a slow taper and justified delayed escalation to azathioprine at the time of relapse rather than earlier initiation of stronger rescue immunosuppressants.

### 2.4 Disease course and relapses

**Figure 3:**
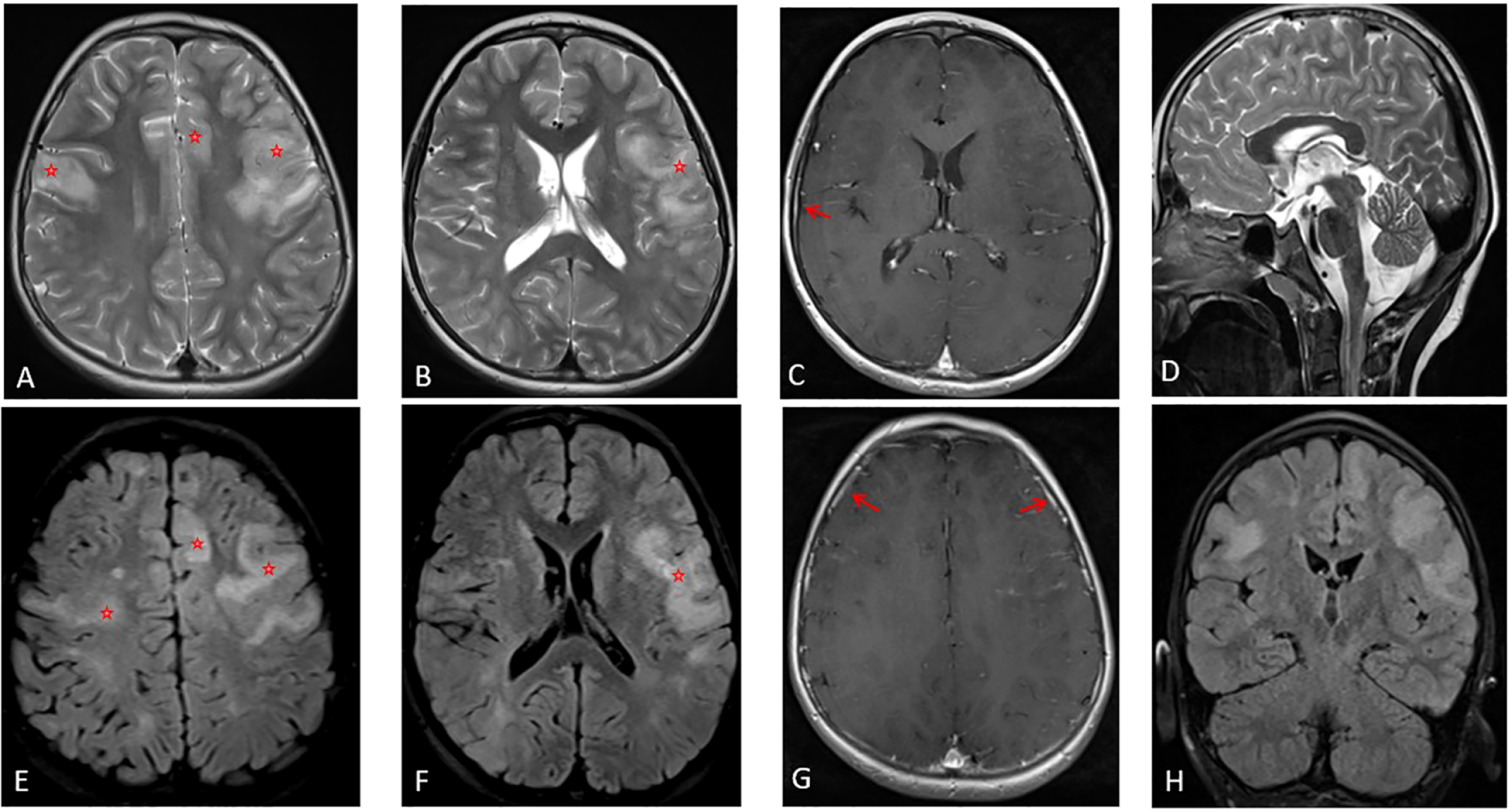
Cerebral MRI findings. Axial T2-weighted **(A, B)**, axial FLAIR **(E, F)**, and coronal FLAIR **(H)** images show bilateral cortical and subcortical hyperintensities with cortical swelling in the frontal and parietal lobes. Patchy contrast enhancement is visible on axial T1-weighted images with gadolinium **(C, G)**. Sagittal T2-weighted **(D)** demonstrates the absence of the previously reported diencephalic and brainstem hypersignal.

In January 2025 (fourteen months after the first episode), the patient presented with focal motor seizures on the left side, followed by rapidly progressive speech disturbances with a lack of words and slurred speech, with no fever or headache. His neurological examination revealed Broca type aphasia along with spastic dysarthria, right hemiparesis, and right upper limb dystonia. No behavior changes or sleep disturbances were noted during this episode. Disease severity was assessed using the Clinical Assessment Scale in Autoimmune Encephalitis (CASE), with a score of 9/27 at relapse indicating severe neurological involvement. Interictal EEG revealed generalized background activity slowing with bilateral frontal epileptiform discharges. A follow-up brain MRI revealed bilateral cortical/subcortical hyper intensities involving the frontal, parietal and temporal lobes (**Figure 3**). This clinical and radiological presentation was suggestive of a MOGAD- associated Cerebral Cortical Encephalitis (CCE) phenotype (**Table 1**). Antibody screening revealed persistent dual positivity, with anti-NMDAR antibodies detected in both serum (2+) and CSF (2+), and anti-MOG antibodies detected in serum (1:100) but absent in CSF. He received intravenous methylprednisolone at the dose of 30 mg/kg/day during 5 days, followed by five courses of plasma exchange with clinical improvement mainly of his speech disturbances. The patient was kept on oral prednisone at the dose of 1 mg/kg/day with a minimal duration of six months, with an oral immunosuppressant drug (azathioprine at the dose of 3mg/kg/day). He was discharged with a speech therapy program, with a CASE score improving to 6/27.

**Table 1:** Timeline of the development of symptoms, key findings from imaging and laboratory tests, diagnosis and treatment.

Date/Event	Clinical Manifestations	Phenotype	Brain MRI findings	Biological screening	Management	Outcome	CASE score
January 2024/First episode	Sleep disorders (daytime drowsiness, narcolepsy) Neuropsychiatric disorders (disinhibition) Multiple cranial nerve palsy Hyperkinetic movements (generalized chorea + cerebellar ataxia)	MNOS	Diencephalic hyperintensities extending to the brainstem Bilateral optic nerve, chiasma and optic band hyperintensities Patchy contrast enhancement in basal ganglia	-Serum: dual positivity of anti-MOG ++ and anti-NMDAR antibodies ++ -CSF: oligoclonal bands and dual positivity of anti-MOG ++ and anti-NMDAR antibodies ++	-Acute management: Intravenous Methylprednisolone (30mg/kg/day for 5 days) with oral tapering Intravenous IgIV (six courses within six months) -Maintenance therapy: none -Symptomatic treatment: motor rehabilitation	Clinical and radiological improvement	9
August 2024/Remission from first relapse	Persistant left third cranial nerve palsy Improvement of sleep and neuropsychiatric disorders		Attenuation of diencephalic and brainstem hyperintensities with no additional anomalies	Serum: anti-MOG antibody positivity + (Anti-NMDAR was not tested)	-Slow tapering of oral corticosteroids over nine months -No maintenance therapy		3
January 2025/ Second episode	Speech disturbances Focal motor seizures	MOGAD (Cerebral Cortical Encephalitis)	Bilateral cortical subcortical hyper intensities, with cortical swelling, in the frontal and parietal lobes with a patchy contrast enhancement	-Serum: dual positivity of anti-MOG ++ and anti-NMDAR antibodies ++ -CSF: oligoclonal bands and positivity of anti-NMDAR antibodies ++	-Acute management: Intravenous Methylprednisolone (30mg/kg/day for 5 days) with oral tapering, followed by five courses of PLEX -Maintenance therapy with Azathioprine (3mg/kg/day) -Symptomatic management: antiseizure treatment (valproate) and speech therapy	Significant improvement after PLEX	9→6 on discharge
May 2025/ Four-month post-relapse follow-up	Mild language impairment, mild dyskinesia, gaze paresis	MOGAD	–	–	Ongoing oral prednisone and azathioprine		3/27

### 2.5 Long- term outcome

At the four-month follow-up after the last relapse, the patient showed neurological improvement, with a residual CASE score of 3/27, reflecting mild language impairment, mild dyskinesia, and gaze paresis. Biological data are summarized in **Table 2**.

**Table 2:** Serum and cerebrospinal fluid test results.

Parameter (Unit)	Serum levels		
	29-01-2024		Normal range
Leucocytes (G/L)	7.070		(4 – 14.5)
Hemoglobin (g/dl)	12.8		(11.1 – 14.7)
Platelets (G/L)	205		(166 – 463)
Erythrocyte sedimentation rate (ESR) (mm/hour)	H1 = 15 H2 = 35		(< 20)
C-Reactive Protein (CRP) (mg/L)	6		(< 8)
Thyroid-stimulating hormone (TSH) (mIU/L)	1.59		(0.4 – 4)
Sodium (mmol/L)	135		(135 – 145)
Potassium (mmol/L)	4.3		(3 – 5)
Calcium (mmol/L)	1.19		(2.2 – 2.7)
Aspartate aminotransferase (AST) (U/L)	31		(8 – 33)
Alanine transaminase (ALT) (U/L)	19		(4 – 36)
**CSF Biomarkers**	**Results**		
	**29-01-2024**	**24-01-2025**	**Normal range**
White blood cells count (/mm^3^)	1	7	(< 5)
Protein (mg/mL)	0.25	0.27	(0.15 – 0.6)
Glucose (mmol/L)	2.84	5.39	(2.77 – 4.44)
Glucose ratio (CSF/Blood)	0.54		(0.41 – 0.88)
Chloride (mEq/L)	118	119	(110 – 125)
Albumin quotient^*^	3.34	3.90	(< 9)
IgG index^**^	0.86	0.66	(< 0.7)
**Antibodies**	**Results**		
	**29-01-2024**	**24-01-2025**	**Normal range**
Anti-MOG (CBA, IIFT)			
Serum	1:100	1:100	(< 1:10)
CSF	1:100	Negative	(< 1:10)
Anti-NMDAR (CBA, IIFT)			
Serum	2+	2+	(< 1:10)
CSF	2+	2+	(< 1:10)

^*^Albumin quotient =CSF albumin/serum albumin ;

^**^IgG index = [CSF IgG/serum IgG] x [serum albumin/CSF albumin].

## 3 Discussion

We report the first pediatric MNOS case in Africa, presenting uniquely with the simultaneous detection of NMDAR-IgG and MOG-IgG antibodies in both CSF and serum at the time of diagnosis. The present case is distinguished by (1): simultaneous NMDAR-IgG and MOG-IgG positivity at diagnosis (2), delayed seizure onset relative to initial encephalitic symptoms (3), absence of recurrent optic neuritis or core demyelinating events, and (4) persistent dual antibody positivity during relapse.

**Supplementary Table 1** provides a comprehensive overview of the clinical and biological features, number of relapses, and treatments administered in all pediatric MNOS cases reported to date. Unlike typical presentations, this case featured seizures later in the disease course and lacked recurrent optic neuritis or other classic demyelinating events. This observation broaden the phenotypic and immunologic spectrum of MNOS. While autoimmune disorders are a common cause of encephalitis in children, the simultaneous presence of MOG and NMDAR antibodies—resulting in MNOS—is rare, particularly in pediatric populations, and has recently become a focus of increasing research interest (8, 20–22). MNOS has been documented in a number of case reports and cohort studies, both in adults and pediatric patients, suggesting that these two autoimmune disorders can present either simultaneously or subsequently within the same patient (3, 4, 6, 20, 23). Fan et al. reported that 11.9% of children with MOGAD exhibited MNOS (6). Zhang et al. demonstrated that among pediatric cases of anti-NMDAR encephalitis, 16.9% of children tested positive for MOG antibodies, and 20.2% presented with demyelinating lesions (24). To the best of our knowledge, this case represents the 64th reported instance in the pediatric population documented in the literature to date (**Supplementary Table 1**). Age at onset ranged from 2.2 to 16 years, and mean age was 8.6 ± 3.2 years, as observed in the present case (**Supplementary Table 1**). Notably, the patient exhibited overlapping clinical and immunological features consistent with both MOGAD and NMDAR encephalitis.

In a large cohort study by Du et al. included 49 young male patients with a median age of 23 years, presenting with MNOS (25), seizures along with psychiatric symptoms, were the most frequent initial manifestations in this cohort. A meta- analysis by Ding et al. reported that the majority of patients with coexisting anti-NMDAR-IgG and MOG-IgG antibodies presented with encephalitic manifestations during their first episode, with seizures occurring in 51.1% of these cases (5). Interestingly, our patient experienced seizure activity later in the course of his disease, during the second episode. Our case report is consistent with a study by Hou et al., who reported a cohort of 7 pediatric patients with MNOS characterized by abnormal behavior, sleep disturbances and movement disorders (23). In fact, sleep disturbances were a distinctive feature for MNOS in comparison with MOGAD, according to a case series by Kang Q et al. (12). Another distinguishing feature of this case was the absence of recurrent optic neuritis and other core demyelinating events, which are commonly observed in patients with MOGAD, suggesting that MNOS might be a distinct entity where encephalitic features exceed demyelinating manifestations (26, 27). During follow-up, our patient relapsed with clinical and radiological features consistent with a CCE phenotype and was accompanied by persistent dual positivity for both antibodies. Atypical phenotypes have been reported with MOGAD, including CCE with speech disorders occurring in 27.8% of patients (13). Thus, these phenotypes when occurring in MNOS are considered MOGAD-associated,

although extra-limbic cortex involvement has also been reported in anti-NMDAR encephalitis with atypical phenotypes (28). In terms of the radiological pattern of MNOS, although it remains unspecific, features of MOGAD are at the forefront with brainstem and subcortical involvement as a common finding, which was the case of our patient (25). Therefore, patients presenting with an encephalitis phenotype and exhibiting demyelinating lesions on MRI should be tested for both anti-MOG and anti-NMDAR antibodies. Detection of neuronal surface antibodies in serum and/or in CSF is conducted by most clinical laboratories using commercial cell-based assay (CBA, Euroimmun^®^) based on indirect immunofluorescence on fixed and permeabilized NMDAR transfected HEK293 cells (expressing the major target antigen; NR1 receptor subunit) (IIFA). Similar challenges are present in the detection of MOG antibodies. CBA serum testing method, involving transfected mammalian cells with MOG antigen, is recommended for MOGAD (29, 30). Current evidence indicates that anti- MOG and anti-NMDAR antibodies do not cross-react. Anti-MOG antibodies target the myelin oligodendrocyte glycoprotein specifically located on the outer surface of oligodendrocytes in the central nervous system (31, 32), whereas anti-NMDAR antibodies target the NR1 subunit of N-methyl-D-aspartate receptors predominately on neurons (33). The co-occurrence of both antibodies in some patients therefore most likely reflects overlapping autoimmune processes rather than true immunological cross- reactivity (34). From a technical perspective, cerebrospinal fluid testing for NMDAR abs provides very high diagnostic accuracy, with specificity exceeding 99%, whereas serum testing is less reliable and may yield false positives in patients with neuropsychiatric conditions or even in healthy individuals (35, 36). For MOG abs, optimized cell-based assays achieve up to 98% specificity, but commercial platforms may produce false negatives due to limited antigen coverage or pre-analytical issues such as sample handling (37, 38).

In the present case, NMDAR-IgG and MOG-IgG antibodies were detected in both CSF and serum at the time of diagnosis, a finding considered rare; a meta-analysis of MNOS cases reported only 10% of patients presenting with dual positivity (5). CSF and serum NMDAR antibody titers are typically higher in patients with poor outcomes (as measured by the modified Rankin Scale) (35) and closely correlate with clinical relapses (39), suggesting that fluctuations in antibody levels may reflect changes in disease activity. Similarly, long-term follow-up data indicate that early seroconversion to MOG-IgG negative status is associated with a reduced risk of relapses (30, 40). In our patient, both MOG and NMDAR antibodies remained persistently elevated during the second relapse (especially in serum), indicating ongoing disease activity. Anti-NMDAR-IgG was re-tested at relapse and remained positive in both serum (2+) and CSF (2+).

MOG-IgG serostatus is known to fluctuate, with higher levels during acute attacks and lower or even absent levels during remission, particularly after a monophasic course; therefore, testing is ideally performed during active disease or treatment-free periods to maximize diagnostic yield (41). Anti-NMDAR antibodies may persist for months to years, and although persistent detection at 12 months has been associated with relapse risk in some cohorts, their presence alone does not reliably predict outcomes, limiting the utility of routine serial testing (42). Accordingly, interval-based monitoring is generally not recommended, and antibodies are typically re-tested only when clinically indicated, such as at relapse or new symptom onset. Longitudinal studies in overlapping syndromes have shown that both MOG and NMDAR titers tend to be higher at relapse and decrease with effective immunotherapy, supporting the clinical relevance of titer changes in reflecting disease activity (41, 43). This approach explains why, in our patient, repeat antibody testing was performed only at relapse, reflecting ongoing disease activity and guiding treatment decisions.

Taken together, these findings highlight the importance of periodic monitoring of MOG-IgG and NMDAR-IgG titers to identify patients with divergent clinical trajectories, as in the present case. This approach could deepen our understanding of the disease phenotype, provide valuable prognostic insights, and potentially guide clinical decision-making. Extensive tumor screening in our patient revealed no evidence of tumor, consistent with findings in all reported pediatric cases (**Supplementary Table 1**).

Our patient had an initial favorable outcome and a good response to first line therapies, but he did eventually necessitate additional immunosuppressive therapies, like most reported cases in the literature (5). Second- line immunotherapies, such as rituximab and cyclophosphamide have demonstrated efficacy in reducing relapse rates and improving neurological outcomes in MNOS (1, 3, 42). Compared to isolated anti-NMDAR encephalitis, patients with overlapping demyelinating syndromes often experience a more protracted disease course and poorer prognosis (44). Given the increased relapse risk and clinical complexity, a more aggressive therapeutic approach is often required in MNOS compared to isolated MOGAD (21, 25, 45). However, the optimal long-term treatment strategy for this overlap syndrome remains undefined (46). Evidence-based guidelines tailored to this subgroup are urgently needed to improve outcomes and minimize long-term disability (47, 48). Furthermore, the long-term impact of MNOS on cognitive function, quality of life, and overall prognosis remain poorly characterized (49). The elevated relapse rates seen in overlap syndromes underscore the need for prolonged follow-up and individualized strategies to prevent recurrence.

## 4 Conclusion

We report the first pediatric case of MNOS in the African population, broadening its recognized clinical and radiological spectrum. Unlike typical cases, our patient developed late-onset seizures without recurrent optic neuritis or classic demyelination, highlighting MNOS heterogeneity. The relapse showed a CCE phenotype with persistent dual antibody positivity, indicating a dynamic immunological profile. This case highlights the importance of early diagnosis, thorough immunological evaluation, and prompt intensive immunotherapy within a precision medicine framework to improve patient outcomes.

## Data Availability

The original contributions presented in the study are included in the article/**Supplementary Material**. Further inquiries can be directed to the corresponding author.
