# Supplementary material for: Case Report: Overlap syndrome of anti–NMDA receptor encephalitis and MOG-associated disease in a pediatric patient—literature insights
Source: Front Immunol. 2026 Mar 10;17:1694771. doi: 10.3389/fimmu.2026.1694771 (PMC13008682; doi:10.3389/fimmu.2026.1694771)
Supplement: Supplementary file 1 [file Table1.doc]

**Supplementary Table 1:** Review of pediatric cases of MOGAD and anti-NMDAR encephalitis (MNOS) overlapping syndrome

| Case | Author (Year) Country | Sex/  Sex-ratio | Age (Years)/  Mean age | Clinical features | | Number of episodes | Anti-NMDAR antibody | | | | Anti-MOG antibody | | | | Treatment | | | | |
| --- | --- | --- | --- | --- | --- | --- | --- | --- | --- | --- | --- | --- | --- | --- | --- | --- | --- | --- | --- |
|  |  |  |  |  |  |  | CSF | serum | | | CSF | | serum | |  |  |  |  |  |
| 1 | Titulaer MJ et al. (2014)  Spain | M | 10 | Seizures, fever, headache, hemiparesis, optic neuritis. | | 2 | Positive | Negative | | | 1:160 | | 1:2560 | | St | | | |  |
| 2 |  | F | 4 | Seizures, speech dysfunction, movement disorders, hemiparesis, orofacial dyskinesias. | | 1 | Positive | Ns | | | 1:2 | | 1:1280 | | St, IVIG | | | |  |
| 3 |  | M | 6 | Behavioral abnormalities sleep disturbances. | | 1 | Positive | Negative | | | > 1:5 | | 1:2560 | | IVIG | | | |  |
| 4 | Yokoyama K et al. (2016) Japan | F | 9 | Fever, headache, sleep disturbances, memory impairment, hemiparesis, dysphagia, optic neuritis, visual loss. | | 2 | Positive | Positive | | | Positive | | Positive | | St, IVIG + IVMP | | | |  |
| 5 | Fan S et al. (2018) China | F | 3 | Optic neuritis, memory impairment, demyelination syndrome episode, speech dysfunction, behavioral abnormalities. | | 3 | 1:32 | Negative | | | 1:32 | | 1:100 | | IVIG + IVMP, MMF | | | |  |
| 6 |  | M | 6 | Seizures, speech dysfunction, movement disorders, memory impairment, behavioral abnormalities, consciousness disorders, autonomic symptoms. | | 5 | 1:100 | 1:32 | | | Negative | | 1:100 | | IVIG + IVMP  MMF + RTX | | | |  |
| 7 |  | M | 9 | Seizures, speech dysfunction, memory impairment, behavioral abnormalities, consciousness disorders, optic neuritis, autonomic symptoms. | | 2 | 1:100 | Negative | | | Negative | | 1:100 | | IVIG + IVMP  St + MMF | | | |  |
| 8 | Sarigecili E et al. (2018) Turkey | M | 6 | Behavioral abnormalities, memory impairment, speech dysfunction, movement disorders, sleep disturbances. | | 1 | 1:320 | Ns | | | Ns | | 1:320 | | IVMP | | | |  |
| 9 | Taraschenko O et al. (2019) United States of America (Omaha) | F | 10 | Fever, headache, optic neuritis, visual loss, seizures. | | 4 | 1:1 | Ns | | | Ns | | Positive | | St + IVIG  RTX | | | |  |
| 10 | Ma J et al. (2020) China | M | 12 | Fever, headache, seizures, behavioral abnormalities, sleep disturbances, dysphagia, hemiparesis. | | 2 | 1:32 | 1:100 | | | Ns | | 1:10 | | St  IVMP + IVIG, RTX | | | |  |
| 11 | Wegener-Panzer A et al. (2020) Germany - Austria | F | 14 | Fever, headache, seizures, behavioral abnormalities, sleep disturbances, hemiparesis. | | 3 | Positive | Positive | | | Ns | | 1:640 | | Aciclovir | | | |  |
| 12 |  | M | 10 | Fever, headache, sleep disturbances, seizures, dysesthesias. | | 1 | 1:10 | 1:10 | | | Ns | | 1:320 | | St, IVMP + IVIG, RTX | | | |  |
| 13 | Zhang R et al. (2023) China | M | 16 | Seizures, speech dysfunction, movement disorders, memory impairment, behavioral abnormalities, optic neuritis. | | 3 | 1:10 | 1:10 | | | 1:100 | | 1:320 | | Aciclovir, St, IVMP + IVIG, CTX + OFT | | | |  |
| 14 | Kang Q et al. (2024) China | F | 6 | Seizures, memory impairment. | | 1 | 1:32 | Negative | | | 1:10 | | 1:100 | | IVMP + IVIG | | | |  |
| 15 |  | F | 5 | Seizures, speech dysfunction, behavioral abnormalities, optic neuritis. | | 1 | 1:32 | Negative | | | Negative | | 1:100 | | IVMP + IVIG | | | |  |
| 16 |  | F | 8 | Movement disorders, hemiparesis. | | 2 | 1:1 | 1:320 | | | 1:10 | | 1:100 | | IVMP + IVIG | | | |  |
| 17 |  | F | 12 | Fever, headache, sleep disturbances, movement disorders, optic neuritis, visual loss. | | 1 | 1:10 | 1:32 | | | 1:100 | | 1:3200 | | IVMP + IVIG | | | |  |
| 18 |  | F | 5 | Behavioral abnormalities, movement disorders, optic neuritis. | | 1 | 1:10 | 1:1000 | | | 1:10 | | 1:1000 | | IVMP + IVIG | | | |  |
| 19 |  | M | 12 | Fever, headache, behavioral abnormalities, sleep disturbances, optic neuritis, visual loss. | | 1 | 1:32 | Negative | | | Negative | | 1:100 | | St | | | |  |
| 20 |  | F | 12 | Fever, headache, seizures, sleep disturbances. | | 1 | 1:1 | 1:10 | | | 1:10 | | 1:100 | | IVMP + IVIG | | | |  |
| 21 |  | M | 11 | Seizures, speech dysfunction. | | 1 | 1:1 | 1:10 | | | 1:100 | | 1:100 | | IVMP + IVIG | | | |  |
| 22 |  | F | 3 | Fever, headache, seizures, behavioral abnormalities, memory impairment. | | 1 | Negative | 1:10 | | | Negative | | 1:10 | | IVMP + IVIG | | | |  |
| 23 |  | F | 12 | Behavioral abnormalities, movement disorders, speech dysfunction, sleep disturbances, memory impairment. | | 1 | 1:100 | 1:100 | | | 1:32 | | 1:320 | | IVMP + IVIG | | | |  |
| 24 | Liao D et al. (2024) China | F | 9.3 | Headaches, seizures, behavioral abnormalities, sleep disturbances, consciousness disorders, speech dysfunction, optic neuritis, autonomic symptoms. | | 5 | Positive | Positive | | | Negative | | Positive | | IVMP + IVIG, RTX, St | | | |  |
| 25 |  | F | 12.8 | Fever, seizures, consciousness disorders,  speech dysfunction, movement disorders, sleep disturbances, orofacial dyskinesias, autonomic symptoms. | | 2 | Positive | Positive | | | 1:32 | | 1:10 | | IVIG +IVMP, RTX, CTX | | | |  |
| 26 |  | F | 8.9 | Seizures, headache, movement disorders, abnormal behavior, memory impairment, sleep disturbances, sphincter dysfunction. | | 4 | Positive | Positive | | | Positive | | Positive | | IVMP + IVIG, RTX, St | | | |  |
| 27 |  | M | 9 | Headache, fever, seizures, optic neuritis, consciousness disorders. | | 5 | Positive | Positive | | | 1:10 | | 1:32 | | IVMP + IVIG, RTX, St | | | |  |
| 28 |  | F | 7 | Movement disorders, abnormal behavior, sleep disturbances, speech dysfunction, sphincter dysfunction, hemiparesis. | | 3 | Positive | Positive | | | Ns | | 1:100 | | IVMP + IVIG, RTX, St, MMF | | | |  |
| 29 |  | F | 2.2 | Fever, encephalopathy, speech dysfunction, paralysis. | | 1 | 1:10 | Negative | | | 1:10 | | 1:100 | | IVMP + IVIG | | | |  |
| 30 |  | F | 8.1 | Movement disorders, behavior abnormalities, memory impairment, orofacial dyskinesias. | | 1 | Positive | Negative | | | 1:10 | | 1:100 | | IVMP + IVIG, RTX, St, AZA | | | |  |
| 31 |  | M | 12.3 | Fever, headache, seizures, abnormal behavior, autonomic symptoms. | | 2 | Positive | Positive | | | Negative | | 1:100 | | IVMP + IVIG, RTX | | | |  |
| 32 |  | F | 9.4 | Seizures, encephalopathy, movement disorders, abnormal behavior, optic neuritis. | | 2 | 1:100 | Positive | | | 1:1 | | 1:100 | | IVMP + IVIG, MMF | | | |  |
| 33 |  | M | 10 | Headache, vomiting, lethargy. | | 1 | Positive | Positive | | | Ns | | 1:32 | | IVMP + IVIG | | | |  |
| 34 |  | M | 6.7 | Seizures, consciousness disorders, memory impairment. | | 2 | 1:32 | Positive | | | Negative | | Positive | | IVMP + IVIG | | | |  |
| 35 |  | F | 8.8 | Headache, seizures, optic neuritis, abnormal behavior. | | 4 | 1:1 | Positive | | | Positive | | Positive | | IVMP + IVIG, RTX | | | |  |
| 36-50 | Xue Y et al. (2025) China  *n* = 15 | M/F = 0.5 | 11  [8 – 13] | Behavioral abnormalities  Consciousness disorders  Cognitive distortions  Memory impairment  Sleep disturbances  Speech dysfunction  Seizures  Movement disorders  Autonomic dysfunction  Central hypoventilation  Optic neuritis | 12 (80%)  9 (60%)  12 (80%)  9 (60%)  12 (80%)  13 (86.7%)  8 (53.3%)  11 (73.3%)  7 (46.7%)  1 (6.7%)  3 (20%) | 9 (60%)  patients relapsed | Positive in serum and/or CSF | | | 12 (80%) positive in both serum and CSF  and 3 (20%) positive in serum only | | | | | | IVMP + IVIG  15 (100%)  PLEX 1 (6.70%)  MMF 7 (46.7%)  RTX 7 (46.7%) | |  | |
| 51-63 | Wang X-Y et al. (2024) China  *n* = 13 | Ns | Ns | Seizures  Headache  Fever  Behavioral abnormalities Speech dysfunction  Movement disorders Consciousness disturbance  Cognitive disorders  Sleep disturbances  Visual disorders | 12 (92.3%)  10 (76.9%)  5 (38.5%)  11 (84.6%)  11 (84.6%)  11 (84.6%)  10 (76.9%)  10 (76.9%)  8 (61.5%)  5 (38.5%) | 11  (84.6%)  patients relapsed | Positive | | Ns | | | Ns | | Positive | | | IVMP + IVIG  7 (53.8%)  MMF 5 (38.5%)  RTX 5 (38.5%)  AZA 1 (7.60%)  IVIG 1 (7.60%) |  | |
| **64** | **Our case** | **M** | **7** | **Optic neuritis, visual loss, memory impairment, behavioral abnormalities, movement disorders, sleep disturbances, speech dysfunction, seizures, hemiparesis.** | | **2** | Positive | | Positive | | | **1:100** | | **1:100** | | | **St**  **IVMP + IVIG**  **PLEX**  **AZA** |  | |

Ns: not specified; F: female; M: male; CSF cerebrospinal fluid; MOG: myelin oligodendrocyte glycoprotein; NMDAR: N-methyl-D-aspartate receptor; St steroids; IVIG intravenous immunoglobulin; MMF mycophenolate mofetil; RTX rituximab; IFN-β interferon-β; AZA azathioprine; CTX cyclophosphamide; IVMP intravenous methylprednisolone; OFT ofatumumab; PLEX plasma exchange.
